# Physical Restraint of Older Adults at Home: Preliminary Analysis of a Prospective Cohort Study

**DOI:** 10.1093/geroni/igaa057.2380

**Published:** 2020-12-16

**Authors:** Asa Inagaki, Ayumi Igarashi, Maiko Noguchi-Watanabe, Mariko Sakka, Chie Fukui, Taisuke Yasaka, Masumi Shinohara, Noriko Yamamoto-Mitani

**Affiliations:** The University of Tokyo, Tokyo, Japan

## Abstract

Our study aimed to explore the prevalence and factors of physical restraints among frail to dependent older adults living at home. We conducted an online survey to ask about the physical/mental conditions, demographics, service utilization, and physical restraints of community-dwelling older adults. Either home care nurse or care managers who were responsible for the older adult answered the survey that were conducted at baseline and one month later. We obtained data from 1,278 individuals. Physical restraint was reported for 53 (4.1%) participants. Multiple logistic regression revealed the factors associated with physical restraints at home: having been restrained at baseline, having pneumonia or heart failure, receiving home bathing, or using rental assistive devices were associated with physical restraints at one month. The findings could be used to promote discussion about which services prevent physical restraints and what we should do to support clients and their family to stay at home safely.

